# Extruded Unripe Plantain Flour as an Indigestible Carbohydrate-Rich Ingredient

**DOI:** 10.3389/fnut.2019.00002

**Published:** 2019-02-05

**Authors:** Daniel E. Garcia-Valle, Luis A. Bello-Perez, Pamela C. Flores-Silva, Edith Agama-Acevedo, Juscelino Tovar

**Affiliations:** ^1^Instituto Politécnico Nacional, Centro de Desarrollo de Productos Bióticos (CEPROBI), Yautepec, Mexico; ^2^Department of Food Technology, Engineering and Nutrition, Lund University, Lund, Sweden

**Keywords:** extrusion, dietary fiber, starch, carbohydrates, digestibility

## Abstract

There is a growing interest in the development of functional ingredients, including those with high indigestible carbohydrate content. Unripe plantain flour (UPF) is a source of indigestible carbohydrates, type II resistant starch (RS) in particular. A major drawback of UPF, however, is that its RS content decreases sharply after wet heat treatment. Here, we explore the possibility of preparing an extruded UPF-based functional ingredient that retains limited starch digestibility features and high dietary fiber content. Both an unripe plantain pulp flour (UPFP) and a whole (pulp and peel) unripe plantain flour (UPFW) were prepared, extruded under identical conditions and evaluated for their gelatinization degree, total starch (TS), resistant starch (RS), and total dietary fiber (TDF) content; functional properties, such as pasting profile, water retention capacity, and solubility, and oil absorption index were also analyzed. The extruded functional ingredient was added to a yogurt and the rheological characteristics and *in vitro* starch digestibility of the product were evaluated. The extruded UPFW showed a lower gelatinization degree than the extruded UPFP, which may be due to the higher non-starch polysaccharide content of the former. A high TDF content was recorded in both extrudates (12.4% in UPFP and 18.5% in UPFW), including a significant RS fraction. The water retention capacity and solubility indices were higher in the extruded flours, particularly in UPFW, while only marginal differences in oil retention capacity were observed among the products. The addition of UPFP or UPFW (1.5 g TDF, w/v) to a yogurt did not alter the viscosity of the product, an important characteristic for the consumer's approval. Moreover, the composite yogurt showed a relatively low starch digestion rate. Extrusion of UPFs may be an alternative for the production of functional ingredients with important DF contents.

## Introduction

In the last years, the end-use of plantain and other varieties of banana has increased around the world. Only in the present year, SCOPUS database shows 14 publications on the use of banana flour in foods, composite films, and modifications to improve its functional and physicochemical features. The use of plantain flour aims to diversify the consumption of the fruit, minimizing the large quantities currently lost during conveying, maturation, and commercialization. The use of plantain fruit in the unripe state is an interesting alternative, due to its high indigestible carbohydrate (components of dietary fiber) content, where resistant starch (RS) is the main component (1). There are diverse studies of the use of unripe plantain flour (UPF) in its native state; however, when this flour is incorporated in heat-treated foods, such as bakery products, snacks, and pasta (2–4), the cooking process largely decreases the indigestible carbohydrate content due to RS conversion to digestible starch. However, we have recently shown that the resistant-to-digestible starch conversion degree depends on the food type. For instance, foods with low moisture content, e.g., UPF-containing cookies, exhibit low conversion rate thus retaining a high dietary fiber content (5). Nevertheless, the drawback of the use of UPF in cooked foods can be overcome with the modification of the flour. Hydrothermal treatments, such as heat-moisture treatment and annealing, were used to modify the starch digestibility in UPF. Rodriguez-Damian et al. (6) reported that heat-moisture treatment of UPF increased its RS content to 11% after cooking. Similarly, the RS content in cooked UPF reached 17% after annealing treatment (7). Additionally, esterification of UPF with citric acid preserved its RS content (93.7%) after cooking (8). The above-mentioned studies have focused on the modification of UPF prepared from the pulp of the fruit; however, in order to decrease the production costs and to increase both the yield and dietary fiber content of UPF, the use the whole fruit (pulp and peel) has been suggested. Such an alternative was tested to produce gluten-free spaghetti. Still, no differences were found in the dietary fiber content (≈ 30) of spaghettis elaborated with pulp-derived UPF compared to those made of whole fruit UPF (9).

Extrusion has been used to prepare pre-gelatinized starches that can be solubilized in water at room temperature. The increased solubility and overall digestibility of the pre-gelatinized starch are attributed to the disorganization of starch components in the granular structure, a process that can be enhanced as the gelatinization degree increases in the extruded starch. However, we hypothesized that if UPF is extruded under specific conditions, it can retain high RS content adding to non-starch polysaccharides in the fiber fraction, and may be thus included in foods where further cooking is not necessary, such as yogurt, smoothies, etc. Such a procedure would be in line with the current interest in the development of foods and ingredients with high dietary fiber contents, which derives from the evidences linking diverse health problems, e.g., overweight, obesity, diabetes, cardiovascular diseases, colon cancer, etc., to the generally low consumption of dietary fiber in occidental populations (10).

The aim of this study was to produce a ready-to-use fiber-rich ingredient by extrusion of UPF, and to perform its chemical, functional and physicochemical characterization. The ingredient was also included in a yogurt to evaluate its impact on the rheological and sensory features of the final product.

## Materials and Methods

### Unripe Plantain Flour (UPF)

Unripe plantain fruits were harvested in Tuxtepe, Oaxaca, México. The flours from the pulp and from the pulp and peel were prepared as described by Ovando-Martinez et al. (1) and Patiño-Rodriguez et al. (9).

### Extrusion

UPF from pulp (UPFP) and the whole fruit (pulp + peel) (UPFW) were extruded in a single screw-extruder (Beutelspacher, México, City, México) at a constant rate of 75 rpm. The temperature in the three zones of the extruder (first zone of the barrel, blend zone, and end zone) was kept constant at 50°C, conditions that in preliminary studies resulted in adequate rheological properties without promoting complete gelatinization of starch in plantain flours (unpublished data). Two batches were prepared for each type of flour.

### Differential Scanning Calorimetry (DSC)

Thermal analysis of gelatinization (in excess water) was assessed to determine the temperatures and enthalpy associated to this phase transition. A 2.0 mg sample was mixed with 7 μl of deionized water. The wetted sample was equilibrated for 12 h at room temperature and subjected to the heating program over a temperature range from 20 to 100°C at a heating rate of 10°C/min in a DSC model 2010 (TA Instruments, New Castle, NJ). The temperatures of the phase transition and enthalpy (area under the phase transition curve) were calculated with the software of the equipment.

### Total Dietary Fiber

The total dietary fiber (TDF) content of the raw and extruded samples was determined by the enzymatic method proposed by McCleary et al. (11), which yields DF values including RS, using experimental conditions that resemble the physiological situation. Total dietary fiber (TDF) was also assessed according to AACC method 32-05 (12).

### Total and Resistant Starch

Total starch (TS) was measured according to AACC method 76.13 (12) using a total starch kit from Megazyme (Wicklow, Ireland). Resistant starch (RS) was determined following the AACC method 32–40 (12) with the RS kit (Megazyme, Wicklow, Ireland).

### Pasting Profile

The pasting profile was evaluated during the cooking of the flours in a stress rheometer (Ar-1500ex, TA Instruments, Dallas, TX, USA) using a starch pasting cell (SPC) with a vanned rotor at 500 s^_1^. The temperature profile started with a heating ramp temperature of 5°C/min from 50 to 95°C, holding at 95°C for 10 min, cooling ramp temperature of 5°C/min from 95 to 60°C and finally holding at 60°C for 10 min. The starch concentration in the sample was 8% (dry basis).

### Water Retention Capacity and Solubility

Water retention capacity was determined according to the method of Hallgren (13). Briefly, 5 ml of water were added to 0.25 g UPF samples (raw and extruded) in pre-weighed centrifuge tubes at room temperature and heated at different temperatures (40–80°C) for 15 min, with shaking at 5 and 10 min. The tubes were then centrifuged for 15 min at 1,000 × g, 10 min. The supernatant was decanted, and the tubes were allowed to drain for 10 min at a 45° angle. The tubes were then weighed and the gain in weight was used to calculate percent gain as the water retention capacity. Experiments were performed in duplicate.

### Oil Absorption Index

The method described by Lin et al. (14) was used to determine oil absorption capacity (OAC). UPF (100.0 ± 0.2 mg) was mixed with 1.0 mL of vegetable oil. The mixture was stirred for 1 min with a wire rod to disperse the sample in the oil. After a period of 30 min in the vortex mixer, tubes were centrifuged at 3000 × g and 4°C for 10 min. The supernatant was carefully removed with a pipette and the tubes were inverted for 25 min to drain the oil and the residue was then weighed. The oil absorption capacity was expressed as grams of oil bound per gram of sample on dry basis. Three replicates were performed for each sample. OAC was calculated by equation:

OAC(g/g)=Wr/Wi

Where:

Wr=residue weight

Wi=sample weight

### Application of the Functional Ingredient in a Non-heat Processed Product. Viscosity Measurement in Yogurt

For viscosity measurements, an in-house made yogurt was mixed with plantain flours to yield total fiber concentrations ranging from 0.1 to 1.5% (w/v). Steady shear rate sweep was carried out at 25°C in a stress-controlled rheometer AR1500ex (TA Instruments, New Castle, USA), using the standard concentric cylinders fixture (HA AL CONICAL DIN, inside radius = 13.98 mm, outside radius = 15.19 mm, length = 42.05 mm, GAP = 5,920 μm). An ascendant sweep from 0.1 to 100 s^−1^ was chained.

### *In vitro* Starch Digestion

The *in vitro* digestibility was assessed according to the methodology described by Zheng et al. (15). In summary, a sample equivalent to 100 mg dry starch was weighed in a beaker and artificial saliva containing porcine α-amylase (250 U per mL of carbonate buffer, pH 7) and pepsin (1 mg per mL in 0.02 M HCl, pH 2) was added. The mixture was incubated at 37°C for 30 min, followed by a second digestion step performed with a mixture of pancreatin (2 mg per mL) and amyloglucosidase (28 U per mL), at pH 6.0. Samples were taken at different time intervals (5, 10, 15, 20, 30, 40, 50, 60, 90, 120, 240, and 360 min) and mixed with 300 μL of stop solution (0.3 M Na_2_CO_3_) to prevent further amylase activity in the aliquot. After centrifugation (2,000 g for 5 min), the glucose concentration in the supernatant was determined using a D-Glucose Assay Kit (GOPOD Format from Megazyme, International, Bray, Ireland) according to the supplier instruction. Results are presented as starch hydrolyzed (grams per 100 g dry starch).

### Sensory Evaluation

One hundred untrained judges evaluated the acceptability of the yogurt. Panelists were asked to assess their degree of liking using a 9-point hedonic scale, where 9 = like extremely and 1 = dislike extremely. The sensory evaluation was targeted to flavor, texture, odor and general acceptance of the yogurt, using the questionnaire proposed by Agama-Acevedo et al. (16).

### Statistical Analysis

Results are presented as mean ± SD (standard deviation). Differences among the means obtained in each of the determinations were evaluated by one-way analysis of variance (ANOVA) with a significance level of α = 0.05, followed by Tukey's test using the statistical package Origin Pro 2016 (OriginLab Corporation, MA, USA). Student's *t-*test was used for the statistical analysis of gelatinization degree.

## Results and Discussion

### Degree of Gelatinization

**Figure 1:**
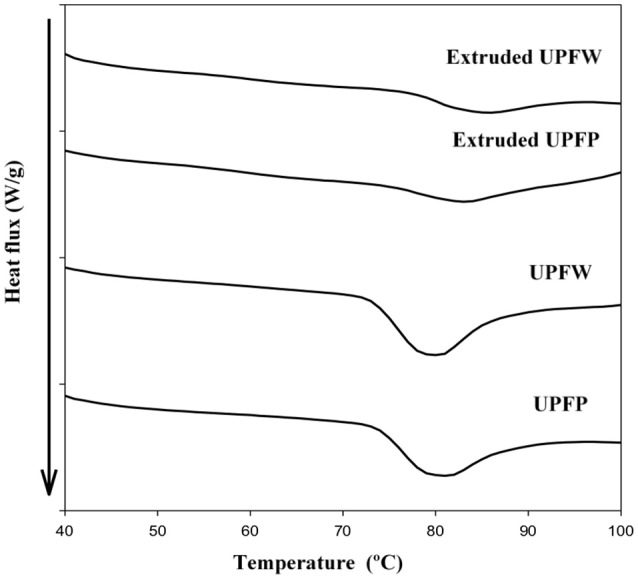
Differential scanning calorimetry of native and extruded flours of unripe plantain pulp (UPFP) and whole fruit (UPFW).

The degree of gelatinization was determined in both extruded samples, the unripe plantain flour of the pulp (UPFP) and that from whole fruits (UPFW). The enthalpy and degree of gelatinization values are shown in Table 1 and the DSC-traces in Figure 1. Only minor differences were observed in the average gelatinization temperatures (Tp) of both flours and extrudates (Table 1), although the extruded UPFP showed a slightly lower value than its UPFW counterpart. This pattern is associated with the higher total starch content in the former sample (Table 2) which is also reflected in the small difference registered in the degree of gelatinization. No information is available in the literature on the degree of gelatinization of extruded UPFs. Usually, extruded flours of different sources show a complete gelatinization, but the conditions used in the current extrusion protocol seem to partially maintain the structural arrangement of starch components of the granules. The difference between both samples can be explained by the dietary fiber content (non-starch polysaccharides) in the flour containing fruit peel, which may have restricted the heat flow transmission during extrusion, as reported in spaghettis elaborated with green banana flour and non-starch polysaccharides (hydrocolloids), resulting in a dense packing and physical entrapment of starch in networks that restrict both the swelling of granules and their accessibility to digestive enzymes (15).

**Table 1:** Temperature (Tp) and enthalpy of gelatinization (ΔH), and gelatinization degree determined by differential scanning calorimetry of native and extruded flours of unripe plantain pulp (UPFP) and whole fruit (UPFW).

**Sample**	**Tp (**°**C)**	**ΔH (J/g)**	**Gelatinization degree (%)**
UPFW	79.6 ± 0.2^a^	8.4 ± 0.2^a^	–
UPFP	78.7 ± 0.2^a^	7.6 ± 0.2^a^	–
Extruded UPFW	85.2 ± 0.2^b^	3.9 ± 0.7^b^	63^a^
Extruded UPFP	83.9 ± 1^b^	2.4 ± 0.2^b^	68^b^

*Values represent mean ± SD of four determinations. Mean values in a column followed by different letters are significantly different (P < 0.05) compared by Student's t-test*.

**Table 2:** Total starch (TS), resistant starch (RS), and dietary fiber (DF) content in native and extruded flours of unripe plantain pulp (UPFP) and whole fruit (UPFW) (g/100 g).

**Sample**	**TS**	**RS**	**DF^**1**^**	**DF^**2**^**
UPFW	74.5 ± 1.1^a^	46.6 ± 0.5^a^	57.2 ± 0.9^a^	12 ± 0.6^a^
UPFP	85.4 ± 0.9^b^	42.8 ± 0.6^b^	49.6 ± 1.0^b^	7 ± 0.5^b^
Extruded UPFW	70.8 ± 0.7^c^	5.8 ± 0.6^c^	18.5 ± 0.6^c^	11.6 ± 0.7^a^
Extruded UPFP	82.5 ± 0.8^d^	4.8 ± 0.7^d^	12.4 ± 0.7^d^	6.8 ± 0.5^b^

*DF^1^ = Method of (11); DF^2^ = Method of 32-05 (12)*.

*Values are reported as mean ± SD of four determinations. Mean values in a column followed by different superscripts are significantly different (P < 0.05)*.

### Starch and Dietary Fiber Content

Total starch (TS) content was higher in the flour of the pulp (UPFP) than in the peel-containing preparation (UPFW), and the same pattern was observed in the extruded samples (Table 2). Extrusion resulted in significantly lower TS contents, which may be related to depolymerization of some amylose chains and formation of transglucosidation products (17), a process that renders alpha-glucans unavailable to amylolytic enzymes (18). The RS in the raw flours was high (around 55% of the TS content) but decreased after extrusion. This reduction in RS content is explained by the above-discussed partial gelatinization of plantain starch, a change that runs in parallel with increased susceptibility to amylolysis (10, 17). However, the RS content remaining in the extruded ingredient obtained from both UPFs (4.8–5.8%) can still be considered high compared with the dietary fiber-associated starch in conventional food items, such as whole wheat bread (1.9–2.8%) and cooked legumes (3–7%) (10, 19).

The total dietary fiber (TDF) content of the flours, determined with a method for products “as eaten” (11), decreased from 49 to 57% in the native preparation to 12–18.5% in the extruded flours (Table 2). In spite of this marked effect of the extrusion treatment, the remaining DF contents were still important. These results may be attributed to the protective effect that non-starch polysaccharides exert against complete gelatinization of starch granules, which allows for a significant retention of the RS component of DF. The DF assessed with the AACC method (12), which measures non-starch polysaccharides and only a minor portion of indigestible starch (i.e., type 3 RS), confirmed that DF in the extruded UPFW is mainly composed of non-starch polysaccharides (11.6%) (Table 2), while the total RS retained (5.8%) fills the gap to the overall 18.5% DF content of the product.

The high DF content recorded in both extruded samples, particularly UPFW, suggests the products' potential as functional ingredients that can be added to a variety of foods, like salads, fruits, smoothies, yogurt, and breakfast cereals. The possible use of the extrudates in other types of food such as salad dressings, emulsions, bakery products and snacks should be further explored.

### Pasting Profile

**Figure 2:**
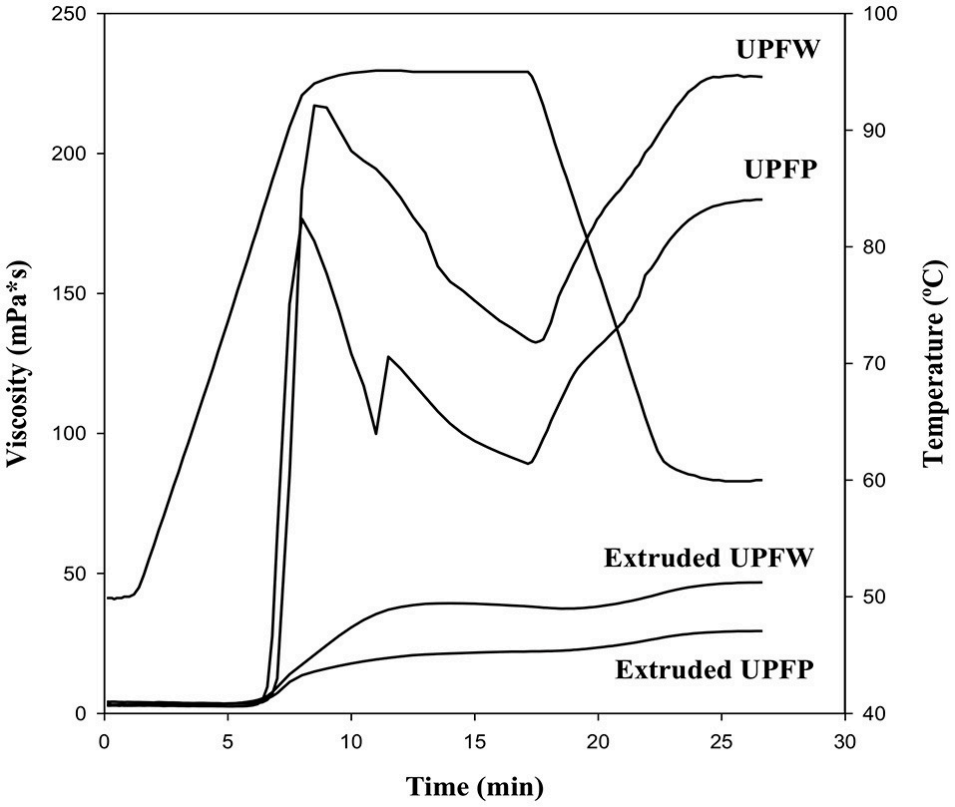
Pasting profile of native and extruded flours of unripe plantain pulp (UPFP) and whole fruit (UPFW).

The pasting profile of both raw flours resembles those exhibited by pure starches (Figure 2), which agrees with the fact that starch is the main component of the flours. The UPFW presented the largest viscosity peak, indicating that the starch granules undergo extensive swelling before they brake, possibly due to the protective effect of the non-starch polysaccharide components of the preparations. The breakdown and setback in both raw flours were similar, indicating that their starch granules broke and reorganized in a similar pattern. UPFW showed the highest final viscosity, which is related to the network produced during cooling, a process that also involves cognate non-starch polysaccharides. The extruded samples developed notably lower viscosity, with no defined viscosity peak, a behavior that is in accordance with the previous heath treatment they underwent. Extruded UPFW exhibited higher maximum viscosity than extruded UPFP. The larger viscosity increase observed for the UPFW extrudate is probably due to the higher concentration and different physicochemical features of the non-starch polysaccharides present in this sample.

### Water Retention Capacity and Solubility

**Figure 3:**
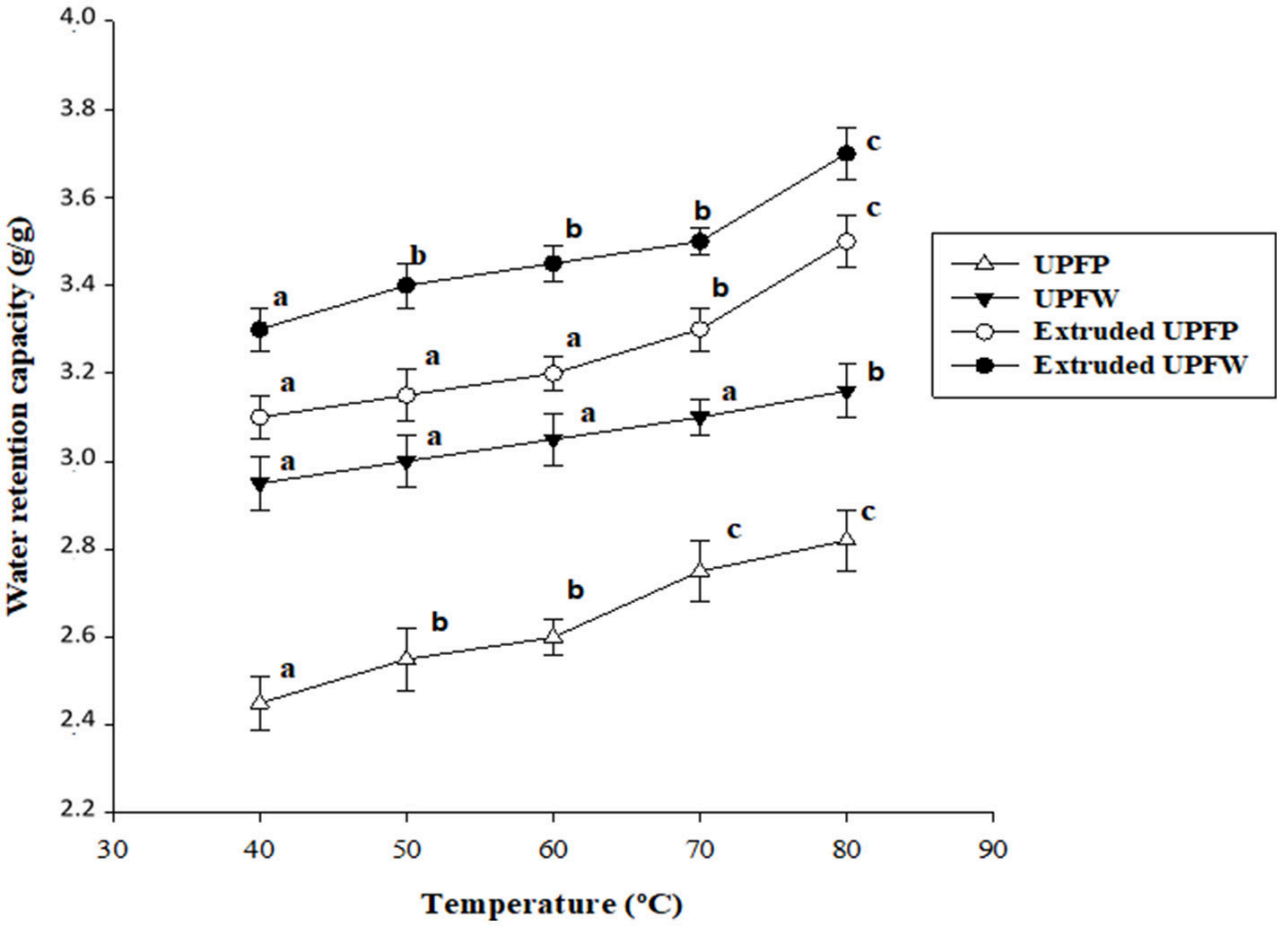
Water retention capacity at different temperatures of native and extruded flours of unripe plantain pulp (UPFP) and whole fruit (UPFW). Different superscript letters on the same curve indicate statistically significant differences (*P* < 0.05).

Both raw flours showed similar water retention capacity (WRC) patterns, i.e., increased WRC concomitant with temperature increments (Figure 3). The higher WRC recorded for UPFW is related to its higher content of hydrophilic components, i.e., starch and dietary fiber (Table 2). The extruded UPFW sample behaved similarly to its raw counterpart, but always with higher WRC values at the different temperatures. Also, the WRC values were higher for the extruded UPFW than for extruded UPFP. Again, the difference between the two extrudates can be related to the higher total starch and DF content of the UPFW extrudate (Table 2). Additionally, as discussed above, UPFW contains a fraction of un-gelatinized starch that may be affected by the additional heat treatment involved in the WRC test, leading to further swelling of those starch granules with the concomitant increase in the WRC. This result reinforces the idea that extruded UPFs may be suitable as RS/DF ingredients in products that do not require further heat treatment before consumption.

**Figure 4:**
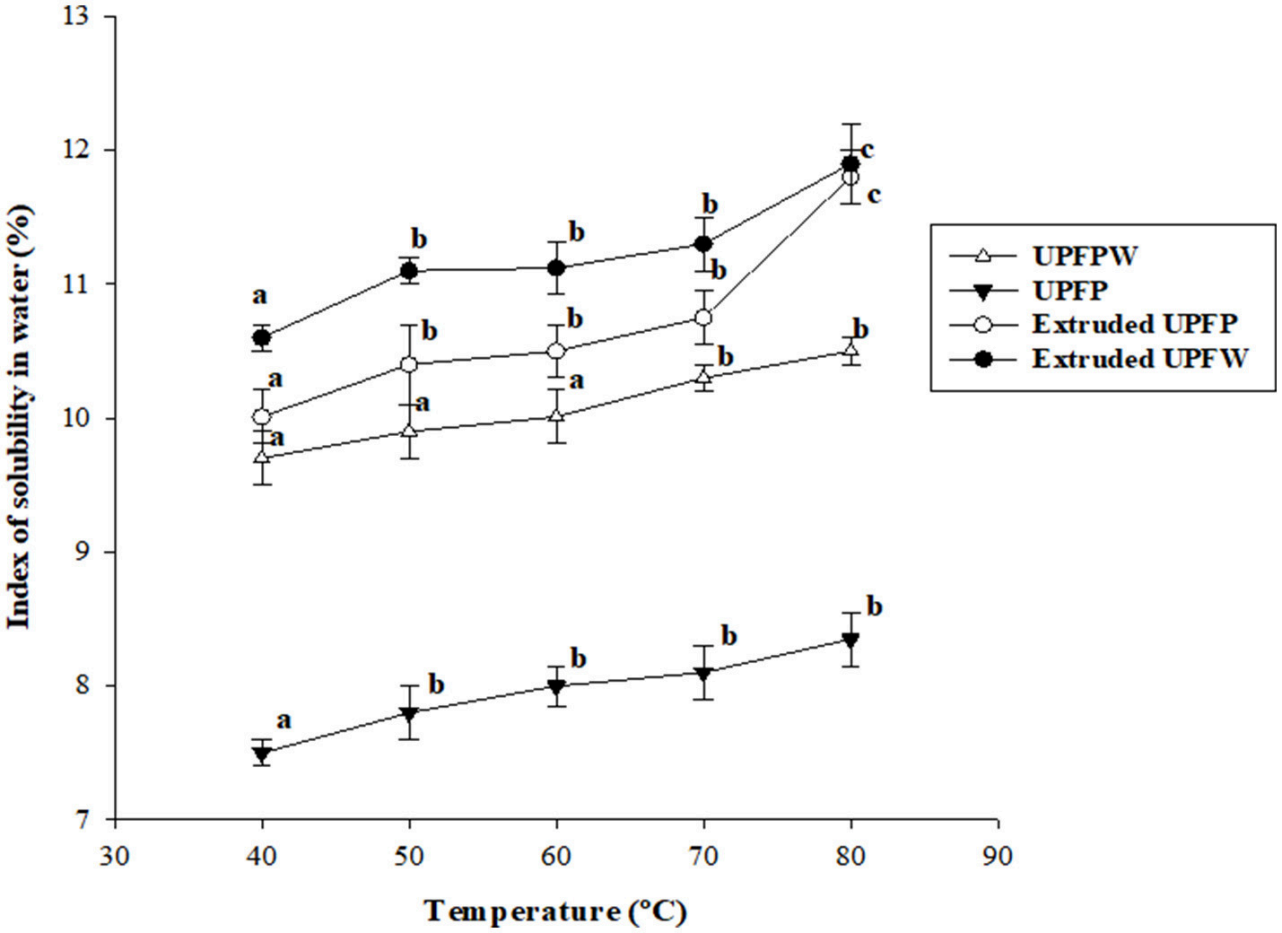
Solubility at different temperatures of native and extruded flours of unripe plantain pulp (UPFP) and whole fruit (UPFW). Different superscript letters on the same curve indicate statistically significant differences (*P* < 0.05).

The temperature-dependent solubility changes of all samples (Figure 4) followed a trend that resembles that of WRC. The extruded UPFP was lower than extruded UPFW, although similar values were determined at the highest temperature tested. The difference between extrudates is related to the higher starch content and degree of gelatinization in UPFW, as starch components (mainly amylose) are solubilized during heating.

### Oil Absorption Index (OAI)

The raw flours showed similar OAI (Table 3). The extruded samples presented a slight increase in the OAI compared to the native ones, and no difference was found between both samples. The higher values observed for the extrudates can be due to the disorganization of starch components during extrusion which results in partial release of amylose chains prone to form inclusion complexes with lipids. The OAIs shows that the starch-rich extruded plantain flours have potential as a fat replacer, given their higher OAI compared to extruded maize flour used to stabilize emulsions (20) and extruded wheat flour used as fat replacer in batter (21). Modified starches have been used in products of reduced-fat content (22), and recently an extruded pre-gelatinized flour was proposed as fat replacer in mayonnaise (20).

**Table 3:** Oil absorption index of native and extruded flours of unripe plantain pulp (UPFP) and whole fruit (UPFW).

**Sample**	**Oil absorption index (g oil/ g solid)**
UPFW	2.55 ± 0.4^a^
UPFP	2.53 ± 0.5^a^
Extruded UPFW	2.68 ± 0.5^b^
Extruded UPFP	2.65 ± 0.7^b^

*Values are reported as mean ± SD of four determinations. Mean values followed by different letters are significantly different (p < 0.05)*.

### Rheological Characteristics of Yogurt

**Figure 5:**
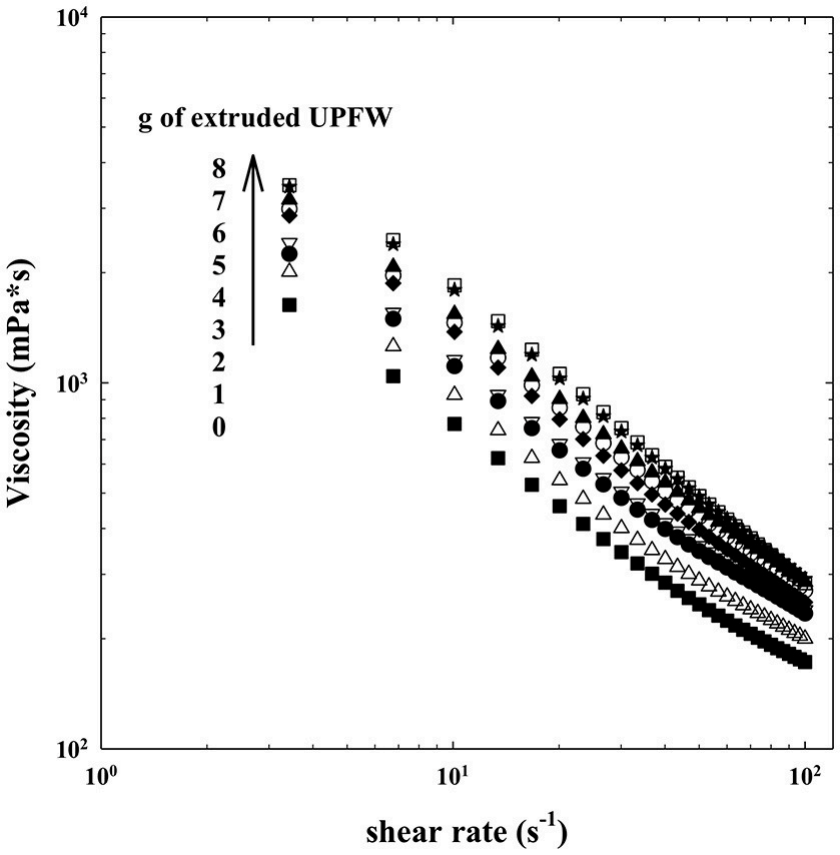
Viscosity of yogurt added with extruded flour of whole unripe plantain (UPFW).

**Figure 6:**
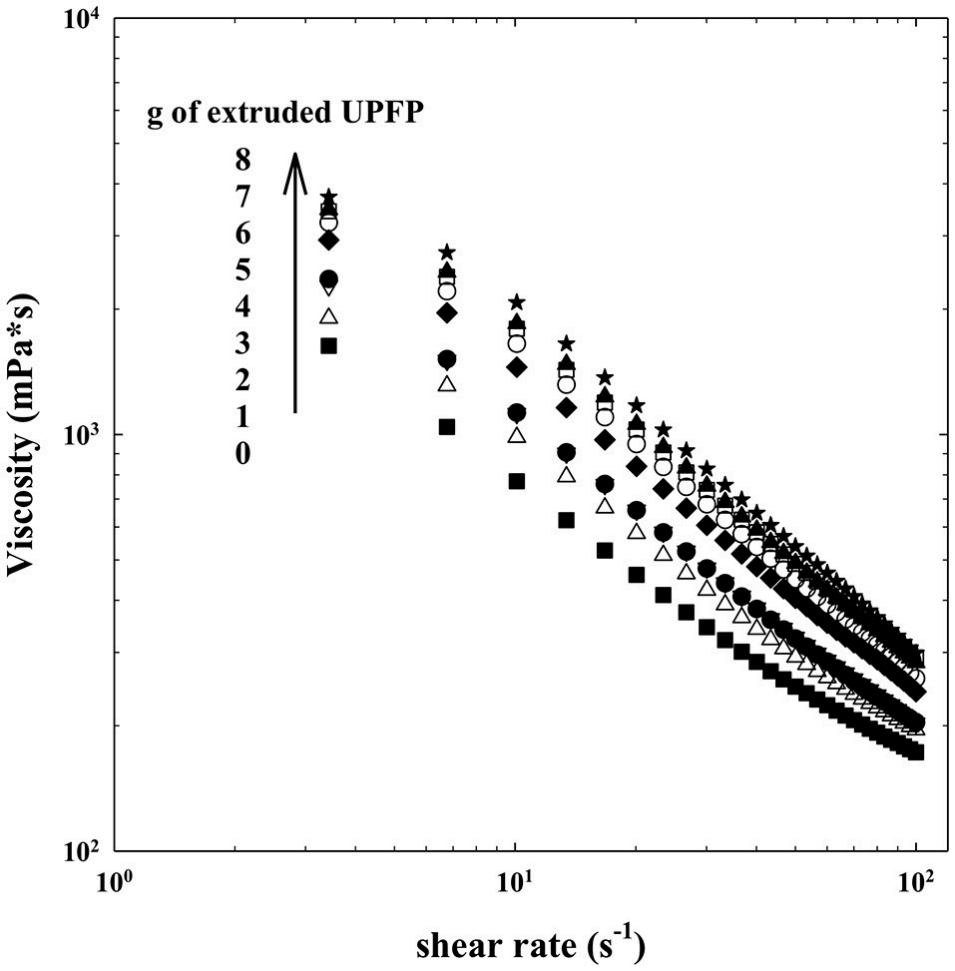
Viscosity of yogurt added with extruded flour of unripe plantain pulp (UPFP).

The effect of the addition of the extruded flours on the viscosity of a yogurt is shown in Figures 5, 6. The viscosity of all samples decreased with the increase in the shear rate, indicating a pseudoplastic behavior. The addition of the extrudates produced a concentration-related increase in the viscosity, without altering the pseudoplastic feature. The impact of the extrudates on viscosity can be attributed to the starch and non-starch polysaccharides present in both samples. The effect on viscosity was higher for the UPFW extrudate. It can be suggested that the fiber components in the whole fruit (e.g., cellulose, hemicellulose) are responsible for the higher viscosity values due to their network-generating ability in this food matrix (23). A similar pattern was found in soy yogurt added with insoluble fiber (24). Power law model was used to fit the experimental data with a *R*^2^ > 0.99 (Table 4). The addition of the extrudates at equivalent concentrations did not change the n value, but the consistency coefficient (*k*) was higher in the yogurt with extruded UPFW, which corroborated the larger viscosity increase this extruded preparation. It interesting to note that here-reported results on the effects of adding unripe plantain-derived flours to yogurts show an inverse pattern when compared to those reported for a yogurt added with resistant starch or β-glucans, where the n value decreased as the polysaccharide concentration increased (25). Evidently, the chemical structure and physicochemical characteristics of the fiber ingredient chosen are important determinants of the sensory-related characteristics of fiber-enriched products.

**Table 4:** Rheological properties of yogurt added with extruded flours of unripe plantain pulp (UPFP) and whole fruit (UPFW).

**g of extruded UPFP/60 mL yogurt**	*K* (mPa*s-1)	***n***	***R*^**2**^**
0 (Control)	3881.50 ± 0.02^a^	0.30 ± 0.0^a^	0.9974
1	4663.37 ± 0.02^b^	0.29 ± 0.0^a^	0.9973
2	5134.52 ± 0.02^c^	0.31 ± 0.0^a^	0.9987
3	5582.13 ± 0.02^d^	0.30 ± 0.0^a^	0.9990
4	6684.97 ± 0.01^e^	0.28 ± 0.0^a^	0.9988
5	6900.80 ± 0.01^f^	0.29 ± 0.0^a^	0.9988
6	7301.29 ± 0.01^g^	0.29 ± 0.0^a^	0.9989
7	8077.92 ± 0.01^h^	0.26 ± 0.0^a^	0.9971
8	8910.45 ± 0.01^i^	0.26 ± 0.0^a^	0.9958
**g of extruded UPFW/60 mL yogurt**			
0 (Control)	3881.50 ± 0.02^a^	0.30 ± 0.0^a^	0.9974
1	5075.75 ± 0.01^b^	0.27 ± 0.0^a^	0.9985
2	5697.70 ± 0.01^c^	0.27 ± 0.0^a^	0.9996
3	5975.85 ± 0.01^d^	0.26 ± 0.0^a^	0.9994
4	7561.22 ± 0.01^e^	0.25 ± 0.0^a^	0.9990
5	8077.92 ± 0.01^f^	0.26 ± 0.0^a^	0.9971
6	8910.45 ± 0.01^g^	0.26 ± 0.0^a^	0.9958
7	9067.75 ± 0.01^h^	0.25 ± 0.0^a^	0.9984
8	9761.12 ± 0.01^i^	0.26 ± 0.0^a^	0.9938

*K, consistency index; n, flow behavior index and R^2^, correlation coefficient. Values with different superscripts within the same column are significantly different (P < 0.05)*.

### *In vitro* Starch Digestion Rate

**Figure 7:**
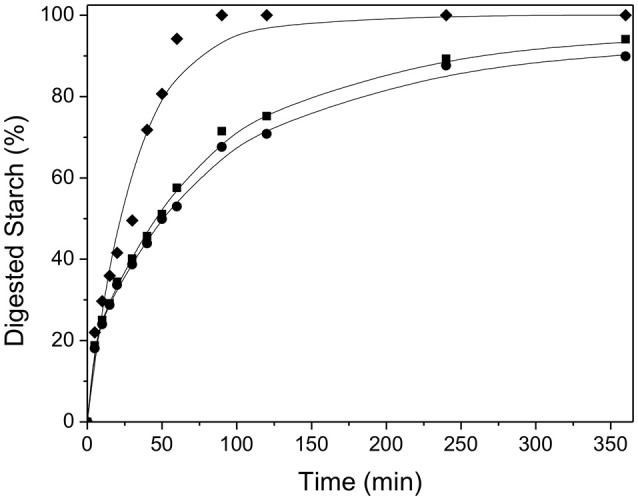
Hydrolysis kinetics and fitting curve for yogurt with the extruded flours of whole unripe plantain (UPFW, •) and flours from the fruit pulp (UPFP, ■). Regular maize starch (gelatinized by 10 min boiling) was used as reference (♦). The lines depict least-squares numerical fitting. Symbols (■, •, ♦) are experimental data.

The *in vitro* starch hydrolysis curves for yogurt added with extruded UPFW and UPFP are shown in Figure 7. Both yogurt preparations showed similar hydrolysis curves with a relatively rapid starch digestion, although it was slower than for the gelatinized starch reference. There may be several reasons for the reduced rate of digestion of the plantain-derived flours. Non-starch polysaccharides present in the food matrix (extruded) can hamper the enzyme (amylase) diffusion and limit the swelling of the substrate (starch granule) resulting in only partial gelatinization; both phenomena retard starch hydrolysis. Also, it has been reported that cellulose and other fibers may inhibit α-amylase activity, attenuating starch hydrolysis (26, 27). Moreover, the extrusion of plantain flours produces partial amylose lixiviation from the granules, and “ghost” granules can produce physical entanglement (low-order starch matrices) that may modulate the hydrolysis by digestive enzymes (28). Since moderate starch digestion rates are considered beneficial in terms of the postprandial metabolic responses to foods (10, 17), the addition of UPFW and UPFP to yogurt may be a positive feature besides increased DF content.

### Sensory Evaluation

The results of the sensory evaluation study (Table 5) showed that control yogurt (no flour added) received the highest score, whilst the yogurt with UPFW had the lowest acceptability, although it was not too different from that of UPFP-containing yogurt. The presence of fruit peel in the extruded flour can be a reason for the low acceptability, as it is well-known that addition of any ingredient in products like yogurts, smoothies, fruits and salads, modify the sensory characteristics and acceptability of the product; however, it should be noted that the appreciation for these products improves after regular consumption and/or addition of flavor (16). On the other hand, the high dietary fiber content in the yogurt, which portraits it as a “healthy food,” can be attractive for consumers with more flexible acceptability criteria. Nevertheless, the sensory features of the yogurt added with the UPFW may be improved, for instance with addition of flavor, an aspect to be explored in future studies.

**Table 5:** Sensory evaluation of yogurt added with extruded flours of unripe plantain pulp (UPFP) and whole fruit (UPFW).

**Yogurt sample**	Overall acceptability^*^
Extruded UPFP	4.7 ± 0.26^a^
Extruded UPFW	3.6 ± 0.21^b^
Control	± 0.32^c^

* *Mean ± SD, n = 100. Means in column not sharing the same letter are significantly different at p < 0.05. Control sample is natural yogurt (no UPF added)*.

## Conclusions

Extrusion of flours from unripe plantain, using either the whole fruit (UPFW) or the pulp (UPFP), produced ingredients rich in indigestible carbohydrates. The extruded UPFW exhibits higher fiber content (including resistant starch), water retention capacity and solubility than the extruded UPFP. The oil absorption index was relatively high in both extrudates. The addition of the extruded flours to a yogurt resulted in slightly increased viscosity, moderate starch hydrolysis rate and somewhat reduced acceptability of the product. Extruded UPFs represent a potential ingredient with nutritional-functional properties that may be used in ready-to-eat processed foods.

## Author Contributions

LB-P and DG-V conceived the study. DG-V, LB-P, PF-S, and EA-A performed the experiments and participated in the acquisition of the data. DG-V, LB-P, PF-S, EA-A, and JT carried out the analysis and interpretation of the data. DG-V, LB-P, and JT drafted the manuscript. All authors read and approved the final version of the paper.

### Conflict of Interest Statement

The authors declare that the research was conducted in the absence of any commercial or financial relationships that could be construed as a potential conflict of interest.
